# Selinexor potentiates NK cell activation against multiple myeloma

**DOI:** 10.1093/cei/uxag059

**Published:** 2026-09-26

**Authors:** Daniel T Harding, Lara V Graham, Laura G Bartlett, Nancy Gudgeon, Jack G Fisher, Christopher J Walker, Trinayan Kashyap, Charlotte E Brookes, Aidan Haslam, Graham McIlroy, Guy Pratt, Ben M Amram, Georgia Francis, Mark S Cragg, Salim I Khakoo, Sarah Dimeloe, Matthew D Blunt

**Affiliations:** School of Clinical and Experimental Sciences, University of Southampton, Southampton, UK; School of Clinical and Experimental Sciences, University of Southampton, Southampton, UK; School of Clinical and Experimental Sciences, University of Southampton, Southampton, UK; Department of Immunology and Immunotherapy, University of Birmingham, Birmingham, UK; School of Clinical and Experimental Sciences, University of Southampton, Southampton, UK; Karyopharm Therapeutics, Newton, MA USA; Karyopharm Therapeutics, Newton, MA USA; School of Clinical and Experimental Sciences, University of Southampton, Southampton, UK; Department of Immunology and Immunotherapy, University of Birmingham, Birmingham, UK; Department of Immunology and Immunotherapy, University of Birmingham, Birmingham, UK; Department of Immunology and Immunotherapy, University of Birmingham, Birmingham, UK; School of Clinical and Experimental Sciences, University of Southampton, Southampton, UK; School of Clinical and Experimental Sciences, University of Southampton, Southampton, UK; Antibody and Vaccine Group, Centre for Cancer Immunology, Faculty of Medicine, University of Southampton, Southampton, UK; School of Clinical and Experimental Sciences, University of Southampton, Southampton, UK; Department of Immunology and Immunotherapy, University of Birmingham, Birmingham, UK; School of Clinical and Experimental Sciences, University of Southampton, Southampton, UK

**Keywords:** multiple myeloma, Selinexor, XPO1, HLA-E, NKG2A, NK cells

## Abstract

Selinexor is a first-in-class selective inhibitor of nuclear export targeting exportin-1 (XPO1) and approved for the treatment of multiple myeloma (MM). Selinexor has previously been shown to enhance natural killer (NK) cell activation against lymphoma cells via disruption of the NKG2A:HLA-E immune checkpoint axis. MM cell lines (L363, U266, MM.1S) and primary myeloma cells isolated from the bone marrow of patients were exposed to selinexor (50–2000 nM) with or without IFNγ or IL-6 to mimic the tumour microenvironment (TME). Surface HLA-E and total HLA class I were quantified by flow cytometry. Immunoblotting and a range of functional assays were used to examine the effect of selinexor on NK cell effector function against MM. Selinexor downregulated surface HLA-E expression on MM cell lines and primary myeloma cells. This improved the activation of NKG2A^+^ NK cells, NK cell-specific lysis of MM cell lines and antibody-dependent cellular cytotoxicity (ADCC) with the therapeutic antibodies daratumumab and elotuzumab. This improvement was also observed in the presence of TME-mimicking signals IFNγ and IL-6. Pre-treatment of MM cells with selinexor for 24 hours prior to daratumumab resulted in optimal ADCC. These data reveal that selinexor selectively disrupts the NKG2A:HLA-E immune checkpoint axis in MM and enhances NK cell activation and ADCC against MM cells. These findings provide a mechanistic rationale for investigating combinations of selinexor with approved therapeutic antibodies in MM and suggest that treatment timing may be an important consideration in the design of combination regimens.

## Teaser

Selinexor downregulates HLA-E on multiple myeloma cells, disrupting the inhibitory NKG2A:HLA-E immune checkpoint and enhancing NK cell activation and cytotoxicity. This effect persists in the presence of IL-6 or IFN? which mimic the myeloma TME, and improves ADCC with daratumumab and elotuzumab, with 24-hour selinexor pre-treatment producing optimal daratumumab-mediated ADCC. These findings support combining selinexor with therapeutic antibodies in multiple myeloma and highlight treatment timing as an important consideration.

## Graphical Abstract

**Graphical Abstract:**
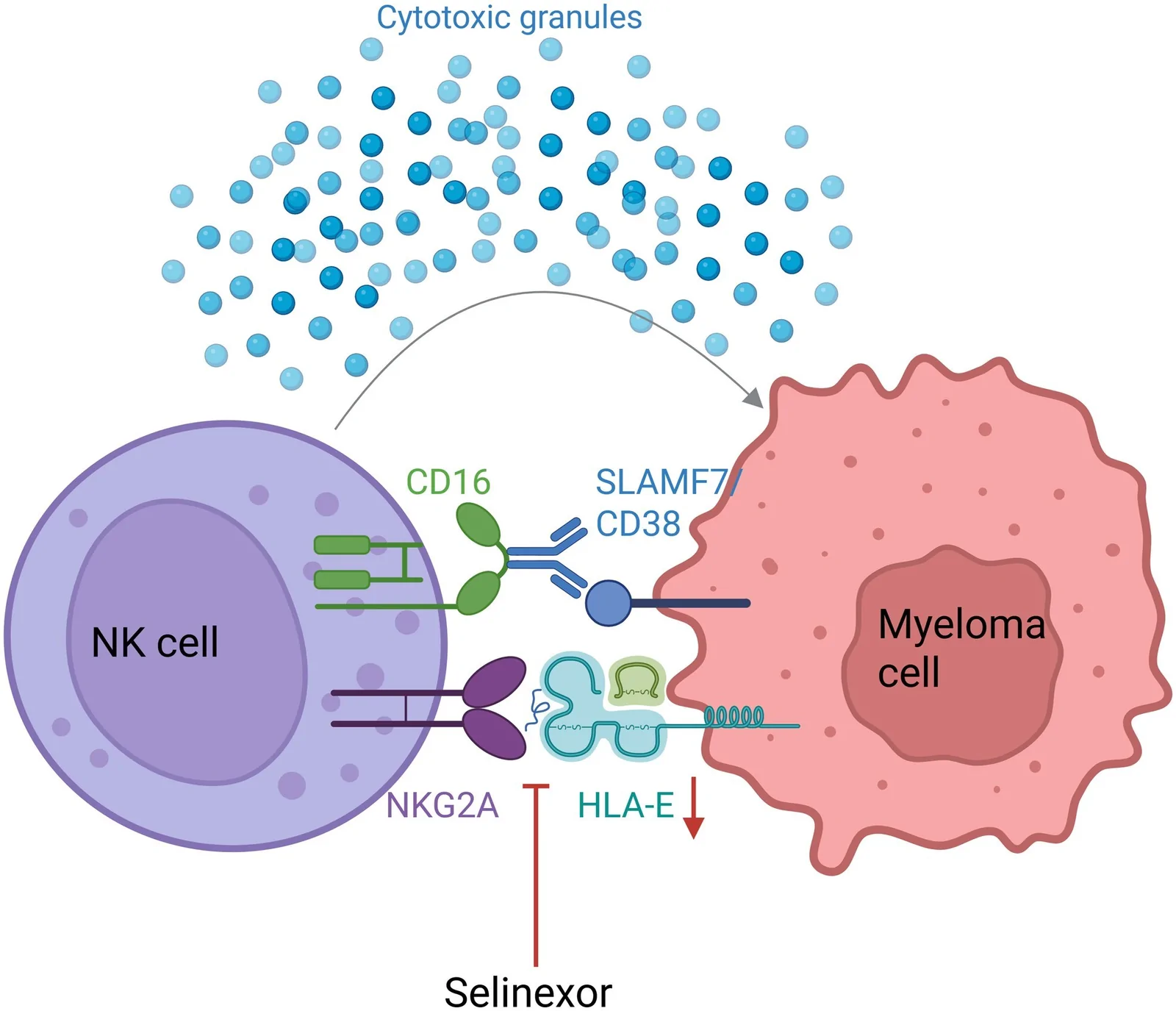

## Introduction

Multiple myeloma (MM) is characterized by clonal expansion of malignant plasma cells within the bone marrow [1]. Despite substantial advances in the therapeutic landscape for MM, including monoclonal antibodies (mAbs), proteasome inhibitors, and targeted therapies, MM remains incurable [1, 2]. The tumour microenvironment (TME) within the bone marrow of patients is characterized by an altered abundance of various cytokines that support MM cell growth. For example, IL-6 produced by bone marrow stromal cells promotes MM plasma cell survival, proliferation, and drug resistance [3]. IFNγ signalling is also enriched in the MM bone marrow and is associated with disease progression [4–6]. This underscores the need for therapeutic approaches capable of enhancing immune responses within this specific TME.

Selinexor is a first-in-class oral selective inhibitor of nuclear export (SINE) that targets exportin-1 (XPO1) and is FDA-approved in combination with bortezomib and dexamethasone for relapsed/refractory MM after at least one prior therapy [7]. By inhibiting XPO1, selinexor prevents nuclear export of tumour suppressor proteins, such as p53, leading to their nuclear accumulation and the apoptosis of malignant cells [8]. Selinexor also induces nuclear accumulation of IκB-α, which leads to inhibition of pro-inflammatory NF-κB signalling. Additionally, XPO1 regulates trafficking of many other signalling proteins, such as STAT1, which is a central mediator of IFNγ signalling via the JAK–STAT pathway [9]. Selinexor has been evaluated in phase II clinical trials in combination with the anti-CD38 mAb daratumumab in relapsed MM patients (NCT06169215, NCT04756401, and NCT04661137) with promising efficacy and safety [10].

Daratumumab and the approved anti-SLAMF7 mAb elotuzumab exert their anti-tumour functions through multiple effector mechanisms [11, 12]. Engagement of the Fc portion of these mAbs with CD16a expressed on natural killer (NK) cells triggers antibody-dependent cellular cytotoxicity (ADCC) against MM cells [13, 14]. NK cells are cytotoxic lymphocytes that are considered the predominant mediators of ADCC in humans, and a low proportion of bone marrow CD16a^+^ NK cells is associated with reduced likelihood of minimal residual disease (MRD) negativity following daratumumab-based therapy in MM patients [15]. NK cell activation is tightly regulated by the balance of multiple activating and inhibitory receptors, and the expression of classical and non-classical MHC I molecules, such as HLA-E, can inhibit NK cell effector function via ligation of the inhibitory killer cell immunoglobulin-like receptors and NKG2A [16]. HLA-E mRNA expression is higher on MM cells compared to normal plasma cells, correlates with more aggressive disease, and contributes to immune escape [17].

We have previously demonstrated that selinexor down-regulates surface HLA-E expression on malignant B cells, and this in turn enhances NKG2A^+^ NK cell activation [18, 19]. We have also demonstrated that selinexor restores NK cell effector function under conditions that mimic the TME in B-cell malignancies with enhancement of anti-CD20 mAb-mediated ADCC [19, 20]. This study therefore sought to assess whether selinexor also modulates NK cell activation and ADCC against MM cells.

## Materials and methods

### Primary samples

Healthy donor peripheral blood mononuclear cells (PBMCs) were obtained from volunteers with ethical approval from the National Research Ethics Committee (REC reference 19/WM/0262 or 24/PR/0245). Bone marrow mononuclear cells were obtained from patients with MM, recruited from University Hospitals Birmingham National Health Service Trust haematology clinics (REC reference 10/H1206/58).

### Cell lines

L363, U266, and MM.1S MM cell lines were used for *in vitro* studies (ATCC). Cells were cultured with RPMI-1640 media (Gibco) supplemented with 10% heat-inactivated foetal bovine serum (Sigma-Aldrich) and 1% penicillin/streptomycin (Life Technologies) (R10) and maintained at 37°C, 5% CO_2_.

### Isolation of mononuclear cells from bone marrow aspirates

Total mononuclear cells were isolated from bone marrow aspirates from patients by density-gradient centrifugation using Ficoll-Paque (Cytiva, catalog #17144003), harvested, washed with phosphate-buffered saline (Sigma, catalog #D8537), counted, and resuspended at 1 million/ml in R10. Patient characteristics are summarized in Supplementary Table S1.

### Isolation and culture of primary NK cells

NK cells were isolated from healthy donor PBMCs using the Miltenyi Biotec human NK cell isolation kit (cat. number 130-092-657). Prior to lysis assays, isolated NK cells were cultured in either R10 with 10 ng/ml IL-15 (R&D Systems) overnight, or MACS medium (Miltenyi Biotec) supplemented with 1% MACS supplement (Miltenyi Biotec), 5% human serum (Sigma-Aldrich), and 500 IU/ml IL-2 (Miltenyi Biotec) at 1 × 10^6^ cells/ml at 37°C in a humidified incubator with 5% CO_2_ for 7–14 days.

### Expression of surface HLA-E and total HLA class I

MM cell lines and primary myeloma cells were pretreated with the caspase inhibitor Q-VD-OPh (QVD, 30 μM; Sigma) for 30 minutes to reduce drug-induced apoptosis. Cells were then incubated with selinexor (50–2000 nM; Karyopharm), ruxolitinib (2 μM; Selleck Chemicals), leptomycin B (LMB, 50 nM; Stratech), or DMSO control as required. After an additional 30 minutes, 10 ng/ml IFNγ or IL-6 (both R&D Systems) was added as required. Following 24-hour incubation at 37°C, cells were stained with total HLA class I-Alexa Fluor® 488 (clone W6/32) and HLA-E-PE/Cy7 or PE (clone 3D12). Primary myeloma cells were also stained with eFluor 780 viability dye (Invitrogen, catalog #13539140), anti-CD38 PE/Cy7(HB-7), and anti-CD138 BV421 (MI15) antibodies (all from BioLegend). Flow cytometry was performed on a BD FACS Aria II (BD Biosciences) or BD LSRFortessa X-20 (BD Biosciences) with data acquired via FACSDiva software (BD Biosciences) and analysed using FlowJo v10.10.0 software (BD Biosciences).

### NK cell degranulation

Target cells (MM.1S or L363) were prepared as above. Healthy donor PBMCs were incubated with 10 ng/ml IL-15 (R&D Systems) overnight and stained with 0.17 μg/ml α-CD107a-eFluor660 (eBioH4A3, Invitrogen) for 10 minutes at room temperature. Stained PBMCs were then added to the target cells at an effector:target (E:T) ratio of 2.5:1 (MM.1S) or 5:1 (L363) for 4 hours at 37°C, with the addition of Golgistop (BD Biosciences) after 1 hour. Data were collected using flow cytometry as above. Gating strategy shown in Supplementary Figure S1. Data presented represent CD107a (%) values minus the no target control.

### NK cell-specific lysis assay

MM cells (MM.1S or L363) were stained with Cell Trace™ Far Red Cell Proliferation Kit (Invitrogen) and were subsequently prepared as above. MM cells were then co-cultured with healthy donor NK cells (either cultured overnight in 10 ng/ml IL-15 or expanded in IL-2 for 7–14 days) at the indicated effector-to-target (E:T) ratio for 24 hours at 37°C. In indicated experiments, daratumumab (10 μg/ml; in-house), elotuzumab (100 μg/ml; Stratech), or relevant isotype control (AT171-2, in-house) was added to MM cells for 20 minutes and washed twice to remove unbound antibody prior to addition of NK cells. After 24 hours, cells were stained with propidium iodide (PI; Invitrogen), and flow cytometry was performed on a BD FACS Accuri C6 Plus (BD Biosciences). Gating strategy shown in Supplementary Figure S2. Data were collected and analysed as above. NK cell-specific lysis was calculated using the following formula:


$$\begin{array}{rl} & \mathrm{NK}\;\mathrm{specific}\;\mathrm{lysis}\\ & =\frac{\left(\text{\%}\;\mathrm{MM}\;\mathrm{lysis}\;\mathrm{in}\;\mathrm{coculture})-(\text{\%}\;\mathrm{spontaneous}\;\mathrm{MM}\;\mathrm{lysis}\right)}{\mathrm{Maximum}\;\mathrm{lysis}-\text{\%}\;\mathrm{spontaneous}\;\mathrm{MM}\;\mathrm{lysis}}\times 100 \end{array}$$

### Immunoblotting

MM cell lines pretreated with the conditions described in the corresponding figure legends were analysed by immunoblotting as previously described [18, 19]. Primary detection antibodies are described in Table 1.

**Table 1:** Primary detection antibodies for immunoblotting.

Target	Clone	Species	Supplier
**STAT1**	42H3	Rabbit	Cell Signaling Technology
**pSTAT1**	D3A4	Rabbit	Cell Signaling Technology
**STAT3**	79D7	Rabbit	Cell Signaling Technology
**pSTAT3**	D3A7	Rabbit	Cell Signaling Technology
**XPO1**	D6V7N	Rabbit	Cell Signaling Technology
**HLA-E**	SAB14011182	Rabbit	Sigma
**β-Actin**	8H10D10	Mouse	Cell Signaling Technology

### Statistical analysis

Statistical analyses were conducted using GraphPad Prism (v10.0.2). Student's *t*-test, one- or two-way ANOVA were used to compare differences between groups when appropriate, as described in the figure legends. Error bars represent the standard error of the mean, and data were considered statistically significant at *P* < 0.05.

## Results

### Selinexor decreases surface HLA-E expression on MM cells and enhances NKG2A^+^ NK cell activation

To test the effect of selinexor on HLA-E expression on MM cells, selinexor (50–2000 nM) was added to MM.1S, U266, and L363 cell lines for 24 hours, and HLA-E and total HLA class I expression were assessed by flow cytometry. HLA-E expression was consistently down-regulated by selinexor to 56–73% of the control at 2000 nM (*P* < 0.01, MM.1S; *P* < 0.0001, U266; and *P* < 0.05, L363) (Fig. 1a–c). In accordance with our previous findings [18, 19], there was no significant change in total HLA class I expression on selinexor-treated cells. Representative raw data can be found in Supplementary Figure S3. In addition, selinexor consistently reduced surface HLA-E expression on primary myeloma cells isolated from the bone marrow (Supplementary Figure S4) of four patients in a concentration-dependent manner to mean of 70% of the control level at 2000 nM (Fig. 1d and e). The observed down-regulation of HLA-E was equivalent for samples from smouldering MM (SMM, open symbols) patients, and a MM patient (closed symbol).

**Figure 1:**
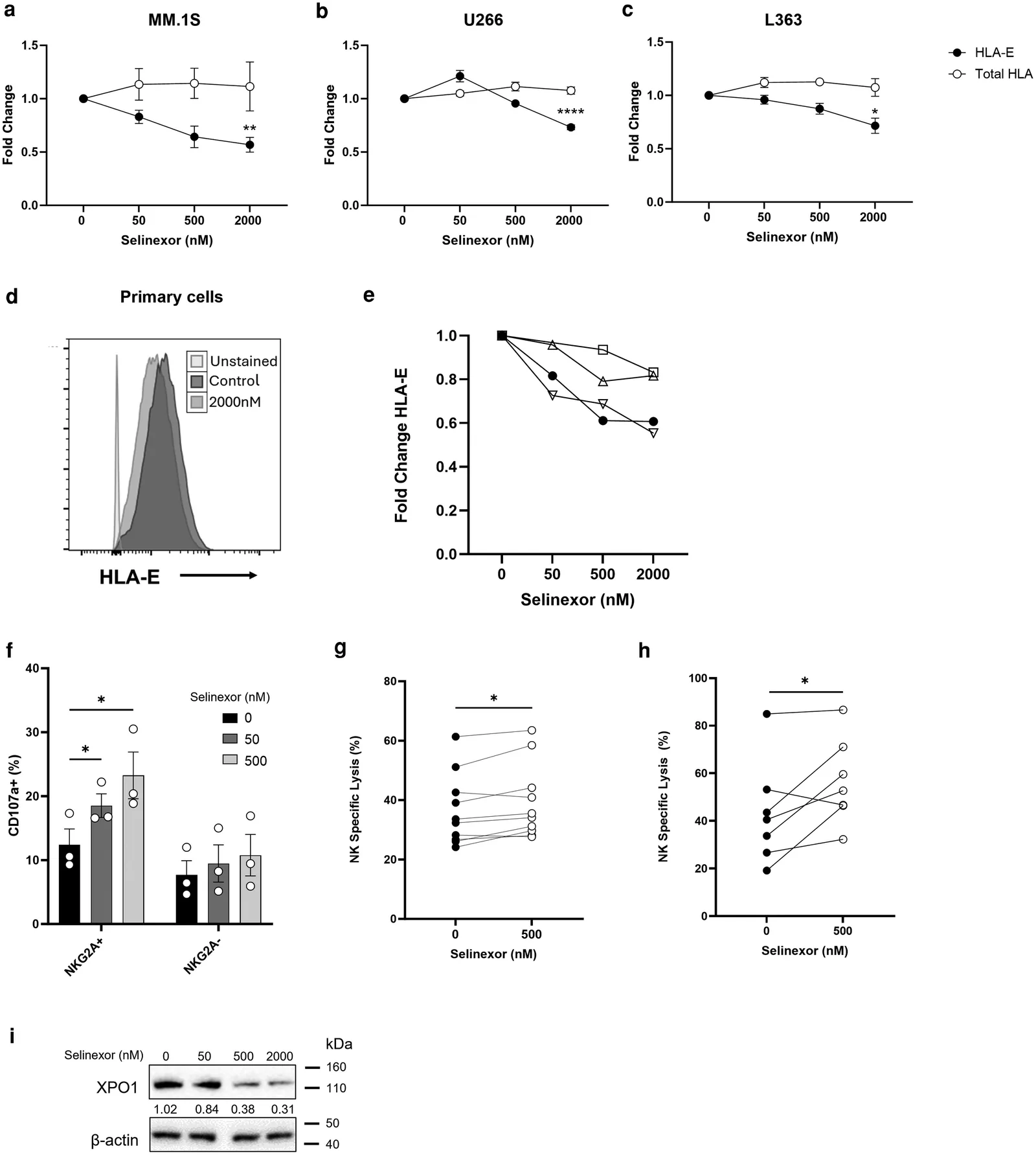
Selinexor down-regulates HLA-E expression on MM cells and enhances NKG2A^+^ NK cell activation. (a) MM.1S (*n* = 5), (b) U266 (*n* = 7), and (c) L363 (*n* = 7) MM cells were treated with selinexor (50–2000 nM) or DMSO control for 24 hours, and surface expression of HLA-E and total HLA class I was analysed by flow cytometry. Shown is mean expression ± SEM calculated as fold change relative to the DMSO control. Representative histograms (unstained, control, and 2000 nM) (d) and summary data (50–2000 nM) (e) showing surface expression of HLA-E on primary myeloma cells treated with selinexor for 24 hours (*n* = 4). MM patient indicated by solid symbol, SMM patients indicated by open symbols. (f) MM.1S cells were treated as above before co-culture with healthy donor PBMCs at a 2.5:1 effector:target ratio for 4 hours. Degranulation (CD107a expression) of NKG2A^+^ and NKG2A^−^ NK cells was assessed by flow cytometry, and data are shown as CD107a expression (%) minus the no target control (*n* = 3). (g) MM.1S (*n* = 9) or (h) L363 MM cells (*n* = 7) were treated with 500 nM selinexor for 24 hours before co-culture with isolated NK cells (cultured in IL-15 overnight) for a further 24 hours at a 2:1 E:T ratio. Lysis of the MM cells was assessed by PI uptake and NK cell-specific lysis is shown. (i) Abundance of XPO1 protein in MM.1S cells treated with 0–2000 nM selinexor assessed by western blotting (representative of two repeats). Numbers represent intensity of the XPO1 bands divided by the β-actin control. Statistical significance determined by two-way ANOVA or *t*-test. *P < 0.05, **P < 0.01, ****P < 0.0001.

Because HLA-E is the ligand for the NK cell inhibitory receptor NKG2A, the activation of both NKG2A^+^ and NKG2A^−^ NK cells against MM.1S cells pretreated with selinexor was evaluated using CD107a staining to assess degranulation (Supplementary Figure S1). Activation of NKG2A^+^ NK cells increased against selinexor-treated MM.1S cells (*P* < 0.05 at 50 and 500 nM) (Fig. 1f), in accordance with previous studies [18, 19]. Furthermore, NKG2A^−^ NK cells did not significantly increase activity in the presence of selinexor (Fig. 1f), indicating that down-regulation of HLA-E by selinexor can enhance NK cell activation by reducing inhibitory signalling through NKG2A. In accordance with enhanced NK cell activation, selinexor pre-treatment increased NK cell-specific lysis of MM.1S cells (Supplementary Figure S5 and Fig. 1g, *P* < 0.05) and L363 cells (Fig. 1h, *P* < 0.05). The caspase inhibitor QVD was added to the MM.1S cells prior to selinexor addition to minimize drug-induced apoptosis of the cells in these assays, and we confirmed that the down-regulation of HLA-E by selinexor is evident in both the presence and absence of QVD (Supplementary Figure S6). Finally, the degradation of XPO1 following selinexor treatment was confirmed by immunoblotting (Fig. 1i). Overall, these data indicate that selinexor enhances NK cell activation against MM cells via disruption of the NKG2A:HLA-E axis.

### Selinexor reduces surface HLA-E expression and increases NK-specific lysis in the presence of IL-6

We have previously shown that signals mimicking the TME in B-cell malignancies (IL-4 and CD40 ligand) can inhibit NK cell function via the HLA-E:NKG2A axis, and that this can be overcome by selinexor [19, 20]. Interleukin-6 (IL-6) is a pro-inflammatory cytokine implicated in promoting MM tumour cell proliferation and survival in the bone marrow [3] and has been shown to up-regulate HLA-E on the surface of senescent fibroblasts *in vitro* [21]. We therefore tested the effect of IL-6 on HLA-E expression on L363 and U266 MM cell lines. In both cell lines, IL-6 did not cause significant up-regulation of HLA-E (Fig. 2a–d). However, treatment with selinexor effectively reduced surface HLA-E expression even in the presence of IL-6 (Fig. 2a–d). In accordance with this, selinexor enhanced NK cell-specific lysis of MM cells in the presence of IL-6 from 8.3 to 22.0% compared to the DMSO control (*P* < 0.05, Fig. 2e).

**Figure 2:**
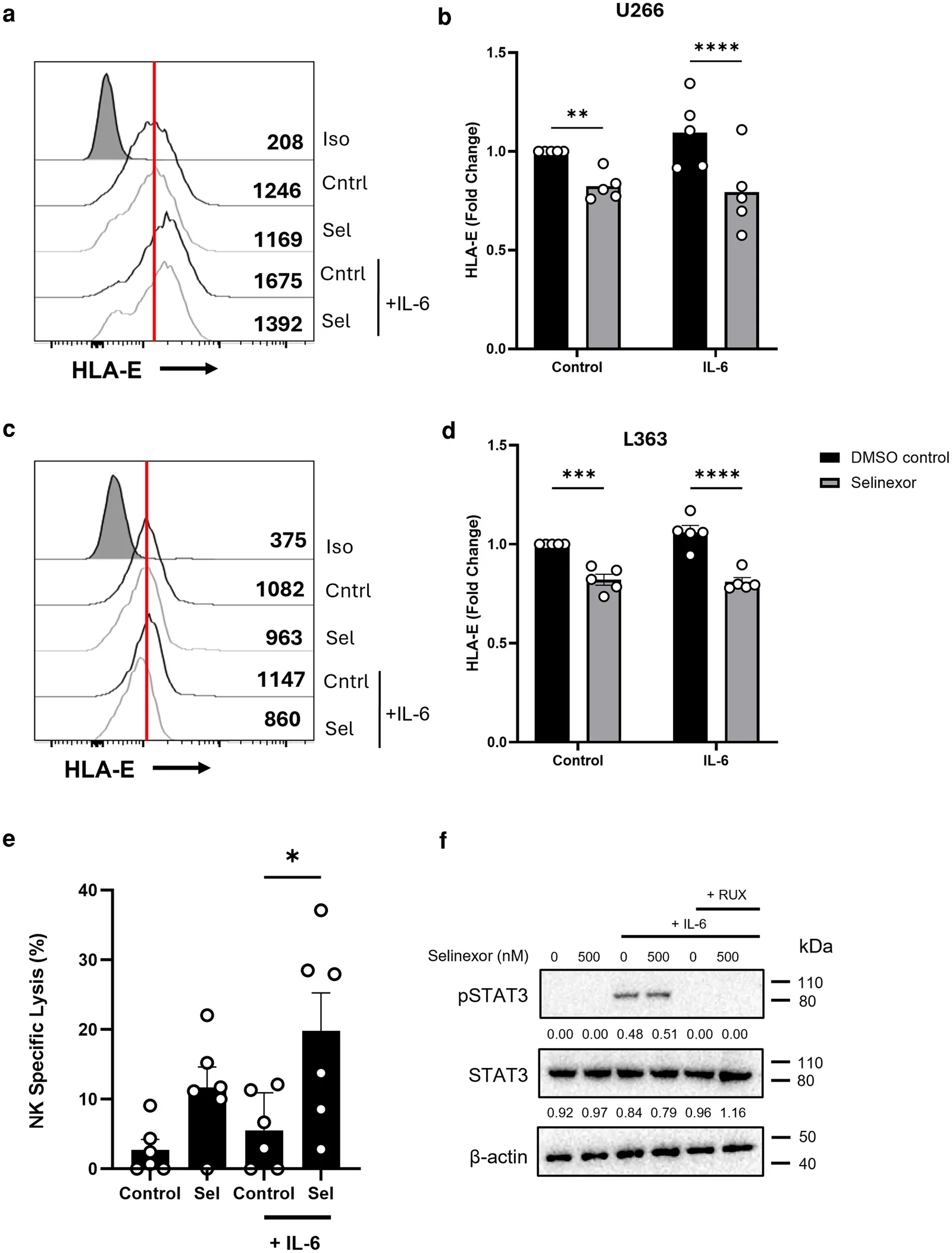
Selinexor down-regulates HLA-E expression on MM cells in the presence of IL-6. (a, b) U266 (*n* = 4) and (c, d) L363 (*n* = 4) MM cells were treated with 10 ng/ml IL-6, 500 nM selinexor, or DMSO control for 24 hours, and surface expression of HLA-E was analysed by flow cytometry. Representative data of U266 cells shown in (a) and data summarized as mean expression ± SEM calculated as fold change relative to the untreated DMSO control in (b) and (c). (e) L363 cells were treated with 10 ng/ml IL-6, 500 nM selinexor, or DMSO control for 24 hours before co-culture with IL-2 expanded NK cells for a further 24 hours at a 1:1 E:T ratio. Lysis of the L363 cells was assessed by PI uptake and NK cell-specific lysis is shown (mean ± SEM, *n* = 4). (f) Phosphorylation of STAT3 (pSTAT3) in L363 cells treated with 10 ng/ml IL-6, 500 nM selinexor or DMSO control was assessed by western blotting (representative of four independent experiments). Ruxolitinib (2 μM) was used as a positive control for inhibition of STAT3 phosphorylation. Numbers represent intensity of the bands divided by the β-actin control. Statistical significance determined by two-way or one-way ANOVA. *P < 0.05, **P < 0.01, ****P < 0.0001.

IL-6 activates the JAK/STAT pathway, leading to phosphorylation of STAT3 [3]. Phosphorylation of STAT3 on treatment of L363 cells with IL-6 was confirmed by immunoblotting (Fig. 2f); however, there was no change in pSTAT3 levels by selinexor (Fig. 2f). In contrast, the JAK1/2 inhibitor ruxolitinib, used as a positive control for inhibition of JAK/STAT signalling, abrogated STAT3 phosphorylation induced by IL-6 (Fig. 2f). These data confirm that selinexor down-regulates surface HLA-E expression on MM cells in the presence of IL-6.

### Selinexor inhibits IFNγ-mediated signalling in MM cells and disrupts the NKG2A:HLA-E immune checkpoint in IFNγ-treated MM cells

IFNγ has previously been shown to up-regulate HLA-E expression on MM cell lines via activation of STAT1 [17]. We therefore assessed whether selinexor could down-regulate HLA-E expression on MM cells in the presence of IFNγ. Treatment with 10 ng/ml IFNγ significantly increased HLA-E expression on both L363 (Supplementary Figure S7, Fig. 3a) and U266 (Supplementary Figure S8; Fig. 3b) cells following 24 hours of culture, whereas selinexor decreased HLA-E expression on both IFNγ-treated and untreated cells in a concentration-dependent manner (*P* < 0.01 untreated and *P* < 0.05 IFNγ-treated L363 cells; *P* < 0.01 IFNγ-treated U266 cells). In accordance with Fig. 1, total HLA class I levels on both cell lines remained stable after selinexor treatment in both the presence and absence of IFNγ (Fig. 3a and b). Similarly, IFNγ increased expression of HLA-E on the surface of primary myeloma cells from bone marrow aspirates (*P* < 0.0001), and selinexor down-regulated IFNγ-induced HLA-E expression (*P* < 0.05 50 nM, *P* < 0.01 500 nM, and *P* < 0.001 2000 nM selinexor) (Fig. 3c). In accordance with this, IFNγ reduced NK cell-mediated lysis of L363 MM cells, and this was overcome by selinexor (Fig. 3d).

**Figure 3:**
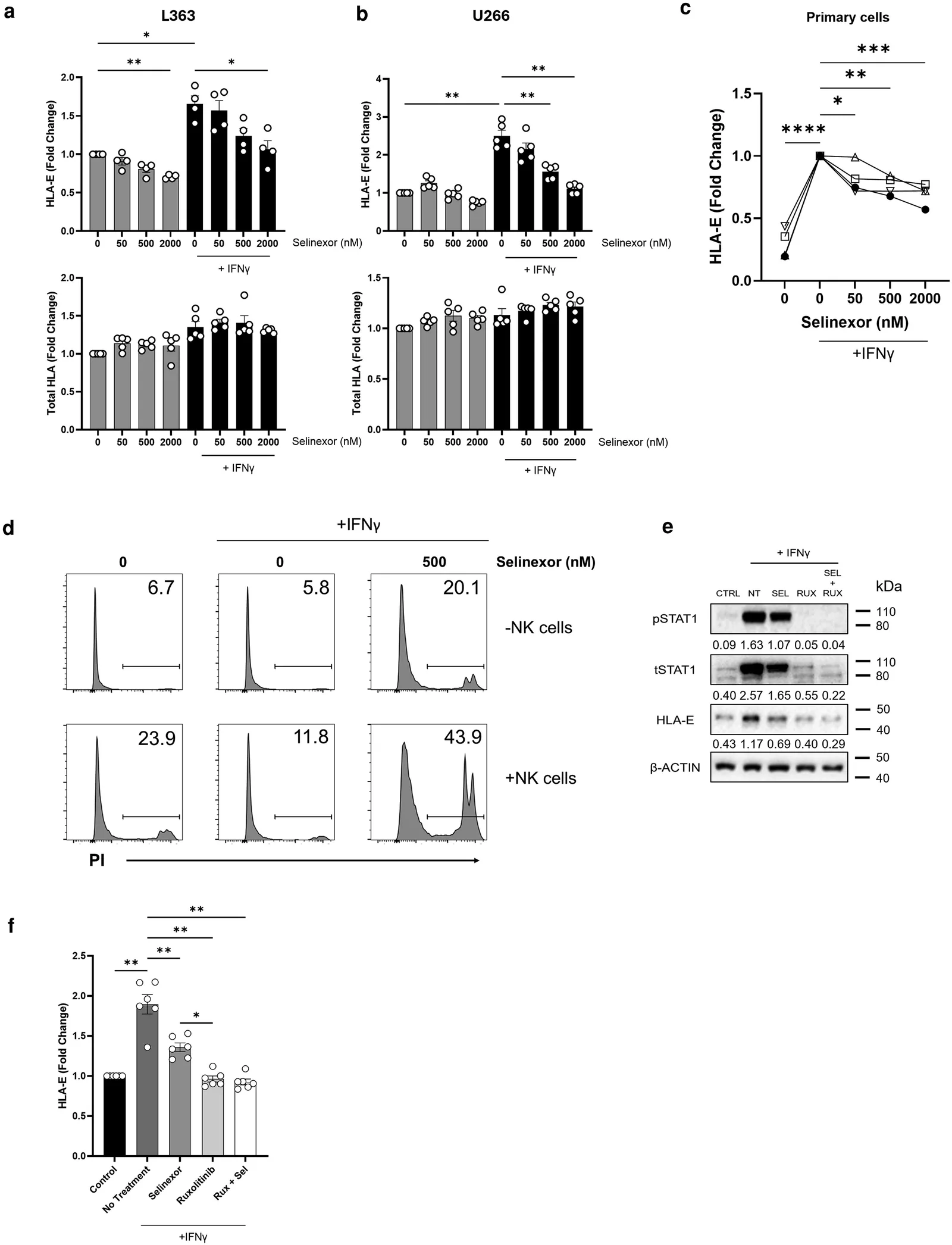
Selinexor inhibits IFNγ-induced HLA-E expression on MM cells and enhances NK cell cytotoxicity. (a) L363 (*n* = 4) and (b) U266 (*n* = 5) MM cells were treated with 10 ng/ml IFNγ, 50–2000 nM selinexor or DMSO control for 24 hours, and surface expression of HLA-E and total HLA class I was analysed by flow cytometry. Shown is mean expression ± SEM calculated as fold change relative to the untreated DMSO control. (c) Surface expression of HLA-E on primary myeloma cells treated with 10 ng/ml IFNγ and selinexor (50–2000 nM) for 24 hours (*n* = 4). SMM patients indicated by open symbols, MM patient indicated by solid symbol. (d) L363 cells were treated with 10 ng/ml IFNγ, 500 nM selinexor, or DMSO control for 24 hours before co-culture with IL-2-expanded healthy donor NK cells for a further 24 hours at a 0.1:1 E:T ratio. Lysis of the L363 cells was assessed by PI uptake. Data are representative of three different donors. (e) Phosphorylation of STAT1 (pSTAT1) in L363 cells treated with 10 ng/ml IFNγ, 500 nM selinexor, or DMSO control was assessed by western blotting (representative of two independent experiments). Ruxolitinib (2 μM) was used as a positive control for inhibition of STAT1 phosphorylation. Numbers represent intensity of the bands divided by the β-actin control. (f) L363 cells were treated with 10 ng/ml IFNγ, 500 nM selinexor, 2 μM ruxolitinib, a combination (Rux + Sel), or DMSO control for 24 hours, and HLA-E surface expression was assessed by flow cytometry. Statistical significance determined by two-way or one-way ANOVA. *P < 0.05, **P < 0.01, ***P < 0.001, ****P < 0.0001.

Because XPO1 can interact with and promote the nuclear export of STAT1 [8], we hypothesized that inhibition of XPO1 with selinexor could down-regulate STAT1 signalling induced by IFNγ. In line with previous reports [17], L363 MM cells treated with IFNγ increased HLA-E protein abundance, and this was decreased by selinexor treatment (Fig. 3e and f). IFNγ increased pSTAT1 and total (t)STAT1 expression (Fig. 3e), consistent with induction of STAT1 signalling by IFNγ [17] and selinexor reduced pSTAT1 and tSTAT1 expression in the presence of IFNγ (Fig. 3e). Ruxolitinib also inhibited STAT1 phosphorylation (Fig. 3e). In addition to selinexor, ruxolitinib also prevented the increase in HLA-E expression on L363 MM cells induced by IFNγ (*P* < 0.01, Fig. 3f).

We next tested the alternative XPO1 inhibitor LMB, which, unlike selinexor, inhibits the function of XPO1 without inducing its degradation [22], as confirmed by immunoblotting (Fig. 4a). LMB significantly reduced HLA-E expression in IFNγ-treated L363 cells to levels equivalent to selinexor (Fig. 4b, *P* < 0.05). In accordance with Figs 1a and 3a, total HLA expression was unaffected by either LMB or selinexor (Fig. 4b), confirming that XPO1 inhibition selectively down-regulates HLA-E in MM cells. We also confirmed inhibition of NKG2A^+^ NK cell activation with IFNγ, and this was recovered by LMB (Fig. 4c and d). Overall, these data reveal that XPO1 inhibition via selinexor or LMB enhances NK cell effector function against MM cells via down-regulation of HLA-E.

**Figure 4:**
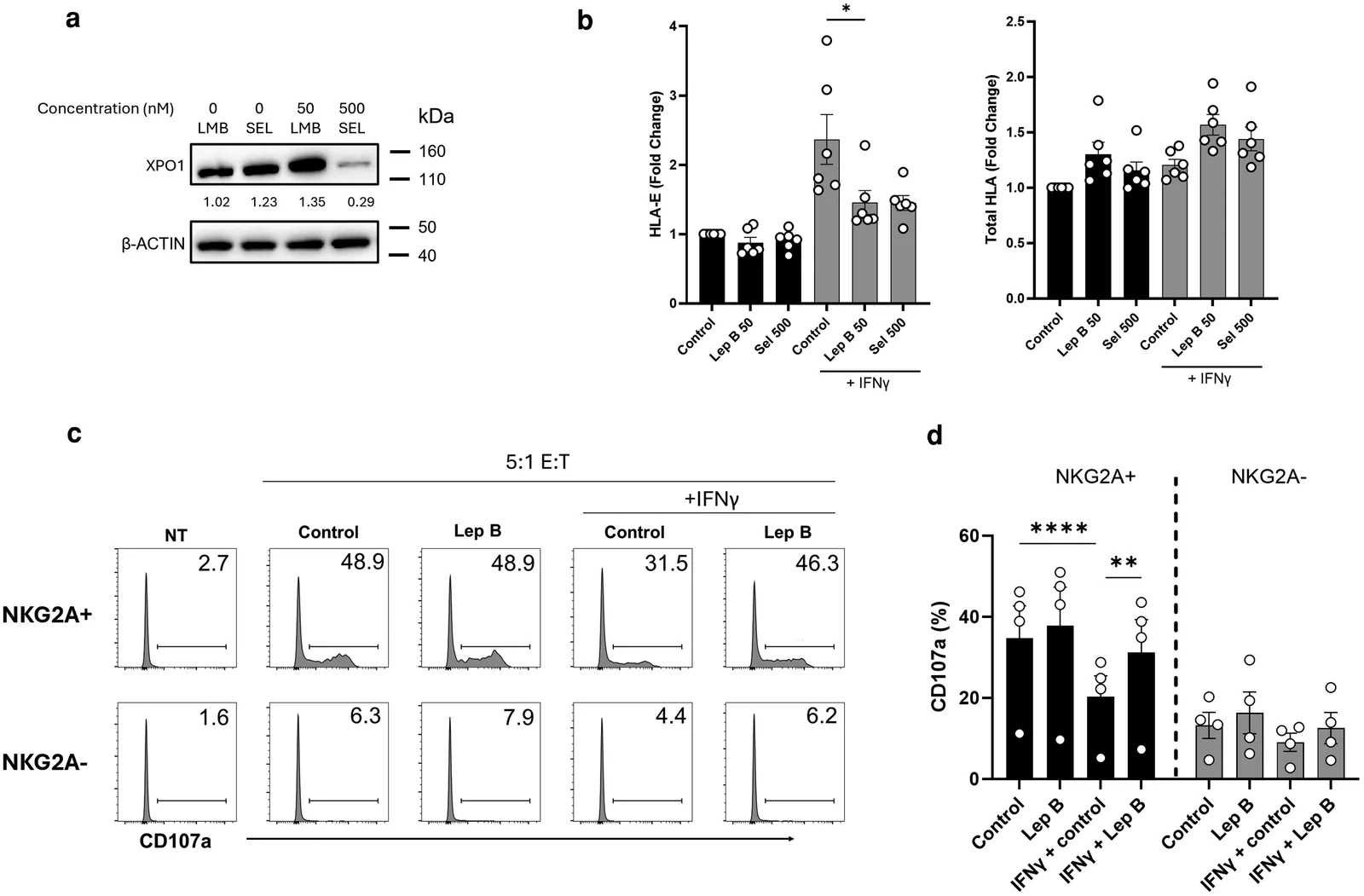
XPO1 inhibition by LMB increases NK cell cytotoxicity against MM cells via disruption of the HLA-E:NKG2A axis. (a) L363 MM cells were treated with 500 nM selinexor, 50 nM leptomycin B (Lep B), or DMSO control for 24 hours, and XPO1 protein abundance was analysed by western blotting. Numbers represent intensity of the XPO1 bands divided by the β-actin control. (b) L363 MM cells were treated as above, and HLA-E and total HLA class I surface expression were measured by flow cytometry. (c, d) L363 cells were treated with 50 nM Lep B or DMSO control for 24 hours before co-culture with healthy donor PBMCs at a 5:1 E:T ratio. Degranulation (CD107a expression) of NKG2A^+^ and NKG2A^−^ NK cells was assessed by flow cytometry. Representative data shown in (c), and data summarized as CD107a expression (%) minus the no target (NT) control shown in (d) (*n* = 4). Statistical significance determined by two-way or one-way ANOVA. *P < 0.05, **P < 0.01, ***P < 0.001, ****P < 0.0001.

### Selinexor improves NK cell-mediated ADCC against myeloma cells in combination with daratumumab and elotuzumab

Selinexor is currently in phase II clinical trials in combination with daratumumab (anti-CD38) for MM, including newly diagnosed MM patients (NCT04782687). Elotuzumab (anti-SLAMF7) is also approved for the treatment of relapsed/refractory MM [14]. We have previously demonstrated that selinexor potentiates NK cell-mediated ADCC against lymphoma cells in combination with anti-CD20 antibodies [18, 20], and so explored if it could also potentiate ADCC with daratumumab and elotuzumab. The combination of selinexor and daratumumab led to significantly increased NK-specific lysis of MM cells compared to treatment with either daratumumab (*P* < 0.01) or selinexor (*P* < 0.05) individually (Fig. 5a and b) in the presence of IFNγ. In addition, selinexor enhanced NK cell-specific lysis induced by elotuzumab in the presence of IFNγ compared to single agent-treatments (Fig. 5c).

**Figure 5:**
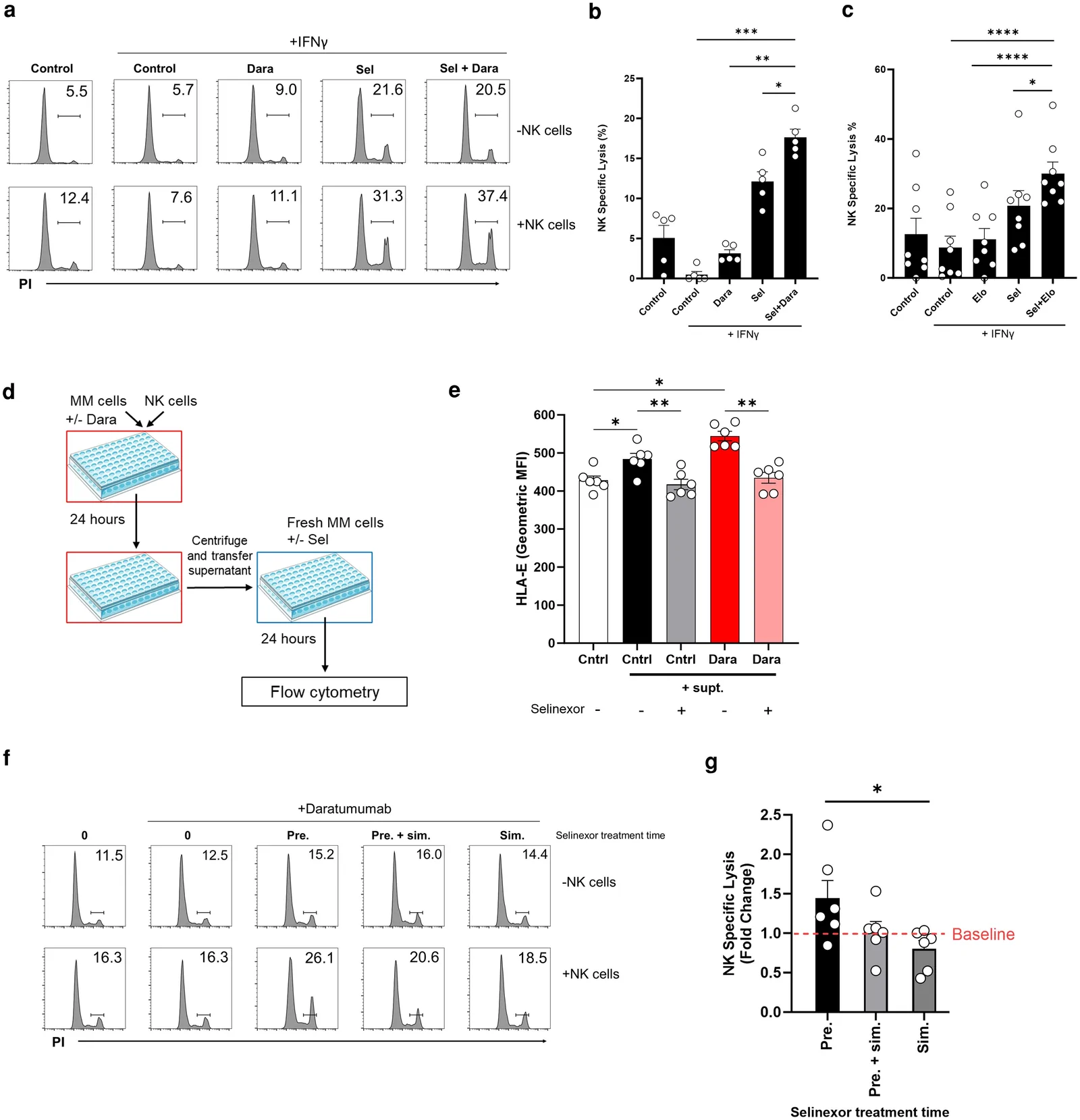
Selinexor potentiates NK cell-mediated ADCC against MM cells in combination with daratumumab and elotuzumab. (a, b) L363 MM cells were treated with 10 ng/ml IFNγ, 500 nM selinexor (Sel), or DMSO control for 24 hours. The next day, 1 μg/ml daratumumab (Dara) or an isotype control was added to the MM cells before co-culture with isolated NK cells at a 0.1:1 E:T ratio for a further 24 hours. Lysis of the L363 cells was assessed by PI uptake. Representative lysis data of IFNγ-treated L363 cells shown in (a) and summarized NK cell-specific lysis data shown in (b) (*n* = 5). (c) Assay performed as in (a) and (b) with 100 μg/ml elotuzumab (Elo, *n* = 6). (d) Schematic showing method for assessing the effect of MM:NK cell co-culture supernatant on surface HLA-E expression. L363 MM cells were co-cultured with 1 μg/ml daratumumab or isotype control, and IL-2 expanded NK cells from healthy donors for 24 hours. Supernatant was collected from the co-cultures following centrifugation, applied to fresh L363 cells and 500 nM selinexor, or DMSO control was added for a further 24 hours. Surface HLA-E expression on the L363 cells was assessed by flow cytometry. (e) Summarized data of HLA-E expression on L363 cells from experiment shown in (d) (*n* = 6). White control (cntrl) bar represents L363 cells treated with DMSO and R10 for 24 hours. (f, g) L363 cells were pretreated (pre.) with selinexor for 24 hours prior to daratumumab, simultaneously with daratumumab (sim.) or both pre. and sim. before co-culture with IL-2 expanded NK cells from healthy donors for a further 24 hours. Lysis of the L363 cells was assessed by PI uptake. Representative lysis data shown in (f), and data summarized as NK cell-specific lysis in the presence of daratumumab shown in (g) (*n* = 6). Dashed line represents baseline NK cell-specific lysis of L363 cells in the absence of selinexor.

ADCC-inducing antibodies have previously been demonstrated to trigger IFNγ secretion by NK cells, which may in turn lead to increased HLA-E expression [23]. We therefore sought to test whether selinexor could inhibit surface HLA-E expression induced by activated NK cells. To do this, supernatants from MM:NK cell co-cultures with or without daratumumab were applied to fresh L363 cells in the presence or absence of 500 nM selinexor or DMSO control for a further 24 hours (Fig. 5d). Application of co-culture supernatants significantly up-regulated HLA-E expression compared to the untreated control, and this was further increased when the co-culture included daratumumab (*P* < 0.05, Fig. 5e). Selinexor down-regulated HLA-E expression induced by co-culture supernatants (*P* < 0.01, Fig. 5e). These data demonstrate that selinexor enhances NK cell-mediated ADCC against MM cells, and that selinexor also impedes the up-regulation of HLA-E expression induced by NK cell activation.

To aid the design of clinical trials that combine selinexor with ADCC-inducing antibodies, we investigated whether the timing of selinexor and daratumumab combination was important for modulation of NK cell-mediated ADCC. L363 cells were either incubated with selinexor for 24 hours prior to daratumumab (pre-treatment), selinexor given at the same time as daratumumab for the 4-hour co-culture only (simultaneous), or selinexor was given for 24 hours prior to re-addition of selinexor with daratumumab (pre-treatment + simultaneous). Selinexor delivery 24 hours prior to daratumumab treatment was the only time point that increased NK cell-specific lysis above the untreated control (Fig. 5f and g). Pre-treatment was also shown to increase NK cell-specific lysis compared with simultaneous delivery (fold change, 1.44 vs 0.80, *P* < 0.05, Fig. 5f and g). These findings indicate that pre-treatment with selinexor prior to the addition of daratumumab is optimal for improving NK cell-mediated ADCC *in vitro*.

## Discussion

Overcoming immune suppression in MM remains a critical barrier to improving the efficacy of immunotherapies and preventing relapse. Previous studies have highlighted that the NKG2A:HLA-E immune checkpoint axis is important in conferring MM cell resistance to NK cell cytotoxicity [17], and blockade [24] or knock-out [25] of NKG2A has been demonstrated to enhance NK cell lysis of MM cells. In this study, we identified that inhibition of XPO1 down-regulated HLA-E at the surface of MM cells in the presence and absence of IL-6 and IFNγ. This led to enhanced activation of NKG2A^+^ NK cells, enhanced NK cell-mediated lysis of MM cells, and potentiation of daratumumab- and elotuzumab-mediated ADCC. Furthermore, pre-treatment of MM cells with selinexor before daratumumab exposure resulted in greater ADCC activity than other schedules tested. Together, these findings are consistent with a model in which selinexor enhances the sensitivity of MM cells to NK cell-mediated cytotoxicity through effects on the NKG2A:HLA-E checkpoint axis, although further studies will be required to establish the relative contribution of this mechanism *in vivo*.

Our study identified that selinexor down-regulates HLA-E at the surface of MM cell lines and primary myeloma samples from the bone marrow of both MM and SMM patients. This was consistent across multiple donor samples, although the magnitude of down-regulation differed between donors. Due to sample limitations, we did not assess whether selinexor could enhance NK cell function against primary myeloma cells; therefore, our conclusions related to NK cell function are based solely on the results from cell lines. In addition, although our data indicate that NK cell activity against MM cell lines is enhanced by selinexor due to disruption of the NKG2A:HLA-E immune checkpoint axis, direct mechanistic confirmation of this could be achieved using an NKG2A blocking antibody. Furthermore, cytotoxicity assays utilizing NKG2A^+^ and NKG2A^−^ NK cell populations separated by fluorescence-activated cell sorting, rather than bulk NK cell populations, could further attribute the enhanced cytotoxicity of MM cells following selinexor treatment to the NKG2A^+^ NK cell population.

The findings of this study align with, and extend, prior reports that XPO1 inhibition with selinexor or LMB potentiates NK cell activity against non-Hodgkin lymphoma and CLL cells [18, 19, 26]. Importantly, total HLA expression was preserved. Loss of classical MHC class I molecules can impair CD8^+^ T-cell anti-tumour responses and immunosurveillance in various types of cancer [27, 28]. Therefore, the selective reduction of HLA-E by selinexor preferentially sensitizes MM cells to NK cell cytotoxicity while preserving ligands necessary for the activation of T cells. The effect of selinexor on T-cell activity was not tested in the current study; however, recent data from a phase I clinical trial (STOMP) in MM patients indicate that selinexor in combination with mezigdomide and dexamethasone enhances CD8^+^ T-cell activation and proliferation [29]. The hypothesis is further validated by up-regulation of proliferation and activation markers on CD3^+^ T cells from patients with MM after selinexor treatment [30].

It is well documented that various cytokines become dysregulated in the MM TME, leading to immune evasion, tumour cell survival, and disease relapse [31, 32]. IL-6 has previously been shown to increase HLA-E expression on fibroblasts [21]; however, IL-6 had no effect on HLA-E expression on MM cells. It is noteworthy that selinexor and other SINE compounds have demonstrated potent anti-inflammatory activity through the inhibition of NF-κB, leading to a reduction in cytokines, such as IL-6, IL-1, TNFα, and IFNγ [33]. In addition, IFNγ has previously been shown to up-regulate HLA-E expression on MM cells via JAK/STAT1 signalling [17]. We further demonstrate that both selinexor and the JAK inhibitor ruxolitinib impair IFNγ-induced HLA-E expression on MM cells via inhibition of JAK/STAT1 signalling. This is particularly important in the context of antibody therapy as activation of NK cells by CD16a-binding antibodies, such as daratumumab, can induce IFNγ secretion by NK cells [20]. The combination of selinexor and ruxolitinib is also being investigated as a therapeutic strategy for the treatment of myelofibrosis (MF). In preclinical studies, this combination inhibited the proliferation of MF stem cells [34]. This combination has also demonstrated clinical efficacy in MF patients and has been associated with a rapid decrease in plasma pro-inflammatory cytokines, including IFNγ and IL-6 [35, 36].

Pre-treatment of MM cells with selinexor significantly enhanced daratumumab- and elotuzumab-mediated ADCC. Selinexor is already in clinical trials in combination with daratumumab (NCT06169215, NCT04756401, and NCT04661137), and this study provides further rationale for incorporating selinexor into antibody-based regimens to increase depth of response and overcome evasion of NK cell effector function. Although selinexor pre-treatment enhanced ADCC, simultaneous treatment did not. This is in accordance with previous findings demonstrating that selinexor pre-treatment, but not simultaneous administration, enhances CAR-T and bispecific antibody T cell-mediated lysis of tumour cells [37, 38]. Additionally, genomic analysis of tumour samples from MM patients revealed that daratumumab and selinexor have anti-correlated mechanisms of resistance, and treatment with a selinexor-based regimen immediately after a daratumumab-containing regimen was associated with improved survival in three independent clinical trials [39]. These data support a treatment strategy involving sequential therapy of selinexor followed by tumour-targeting antibodies, such as daratumumab or elotuzumab. This warrants further investigation to maximize clinical outcomes.

Anti-B cell maturation antigen (BCMA) CAR-T cells are approved for the treatment of relapsed/refractory MM [40], and BCMA targeting CAR-NK cells are currently in clinical trials for MM (NCT06045091). To generate sufficient numbers of cells for adoptive therapy, CAR-NK cells are expanded *ex vivo*, which results in significant up-regulation of NKG2A expression. Furthermore, tumour-infiltrating CD8^+^ T cells and exhausted CAR-T cells have been shown to up-regulate NKG2A expression [41, 42]. These observations suggest that the selinexor-mediated disruption of the NKG2A:HLA-E immune checkpoint identified in MM cells in this study may have broader implications for cellular immunotherapies, including CAR-NK and CAR-T-cell therapies. Further investigation will be required to determine whether modulation of this pathway can enhance the efficacy of these approaches in MM.

## Conclusion

In summary, this study identifies selinexor as a modulator of the NKG2A:HLA-E immune checkpoint axis in MM and demonstrates that selinexor treatment is associated with enhanced NK cell activation and ADCC against MM cells. These findings provide mechanistic support for the combination of selinexor with approved therapeutic antibodies and suggest that disruption of immune escape pathways may contribute to the activity of such combinations. Further studies are warranted to determine the translational and clinical relevance of these findings in patients with MM.

## Supplementary Material

uxag059_Supplementary_Data

## Data Availability

All data generated or analysed during this study are included in this published article [and its Supplementary material], and the raw data will be made freely available upon request.

## Abbreviations:

ADCC: antibody-dependent cellular cytotoxicity
FACS: fluorescence-activated cell sorting
MRD: minimal residual disease
MM: multiple myeloma
NK cell: natural killer cell
SINE: selective inhibitor of nuclear export
TME: tumour microenvironment
