# Supplementary material for: Selinexor potentiates NK cell activation against multiple myeloma
Source: Clin Exp Immunol. 2026 Sep 26;220(1):uxag059. doi: 10.1093/cei/uxag059 (PMC13655613; doi:10.1093/cei/uxag059)
Supplement: Supplementary file 1 [file uxag059_supplementary_data.docx]

| **Supplementary Table 1: Clinical characteristics for patients** | | | | | | | | |  |
| --- | --- | --- | --- | --- | --- | --- | --- | --- | --- |
| **Patient number** | Sex | Age | Disease Stage | PP level (g/L) | Trephine % MMPC | PP type | Β2M (mg/L) | Albumin | Cyto-genetics |
| **QE402** | M | 58 | Myeloma | 39.9 | 35 | IgG kappa | 3.1 | 32 | No high-risk lesions |
| **QE413** | M | 81 | SMM | 15.3 | 20 | IgG kappa | 2.3 | 37 | No high-risk lesions |
| **QE419** | M | 69 | SMM | 16.9 | 15 | IgG kappa | 4 | 39 | IGH::CCND1 only |
| **QE444** | M | 48 | SMM | 19.8 | 10 | IgG lambda | 4.3 | 38 | Not Done |

**Supplementary Figure 1: Gating strategy to assess degranulation of NKG2A+ and NKG2A- NK cells by flow cytometry.**

NK cells (CD56+CD3-) were identified from the lymphocyte and single cell gate. NKG2A+ and NKG2A- NK cells were then gated. The no target (NT) controls in the degranulation assays were used to set the gates for CD107a expression of the NKG2A+ and NKG2A- NK cell populations.

**Supplementary Figure 2: Gating strategy to assess NK cell-mediated lysis of target cells by flow cytometry.**

Target cells were identified as CellTrace^TM^+ (CellTrace^TM^- cells represent NK cells). Dead target cells were identified as propidium iodide+ (PI).

| **MM1S** | |
| --- | --- |
|  |  |
| **U266** | |
|  |  |
| **L363** | |
|  |  |
| **Supplementary Figure 3: Selinexor downregulates surface HLA-E expression on myeloma cell lines.**  MM1S, U266 and L363 MM cells were treated with selinexor (50–2000 nM) or DMSO control (0 nM) for 24 hours. Surface expression of HLA-E and total HLA was analysed by flow cytometry. Mean fluorescence intensity (MFI) values are annotated. Data is representative of 5 (MM1S) or 7 (U266 and L363) repeats. | |

**Supplementary Figure 4: Gating strategy to identify MM cells from primary bone marrow aspirates by flow cytometry.**

**Supplementary Figure 5: Selinexor enhances lysis of MM1S cells by NK cells.**

MM1S cells were treated with 500 nM selinexor for 24 hours before co-culture with isolated NK cells (cultured in IL-15 overnight) for a further 24 hours at a 2:1 E:T. Lysis of the MM1S cells was assessed by propidium iodide (PI) uptake. Data in the presence and absence of NK cells is shown, representative of 10 repeats.

**Supplementary Figure 6: Downregulation of HLA-E expression by selinexor occurs in the presence and absence of QVD.**

MM1S cells were treated with selinexor (50–2000 nM) or DMSO control (0 nM) for 24 hours in the presence or absence of the caspase inhibitor QVD (30 µM). Surface expression of HLA-E was analysed by flow cytometry. An isotype control (iso) is also included to confirm positive staining for HLA-E. Mean fluorescence intensity (MFI) values are annotated. Data is representative of 3 independent experiments.

**Supplementary Figure 7: Selinexor downregulates HLA-E expression at the surface of L363 cells in the presence and absence of IFNγ.**

L363 MM cells were treated with 10 ng/mL IFNγ, 50-2000 nM selinexor or DMSO control for 24 hours and surface expression of HLA-E and total HLA class I was analysed by flow cytometry. Mean fluorescence intensity (MFI) values are annotated. Mean fluorescence intensity (MFI) values are annotated. Data is representative of 7 repeats.

**Supplementary Figure 8: Selinexor downregulates HLA-E expression at the surface of U266 cells in the presence and absence of IFNγ.**

U266 MM cells were treated with 10 ng/mL IFNγ, 50-2000 nM selinexor or DMSO control for 24 hours and surface expression of HLA-E and total HLA class I was analysed by flow cytometry. Mean fluorescence intensity (MFI) values are annotated. Mean fluorescence intensity (MFI) values are annotated. Data is representative of 7 repeats.
